# A Vaccine Meets Its Promise: Success in Controlling Epidemic Meningitis in Sub-Saharan Africa

**DOI:** 10.1093/cid/civ490

**Published:** 2015-11-09

**Authors:** Luis Sambo, Margaret Chan, Steve Davis, Anthony Lake, Seth Berkley, Cyrus Poonawalla, Christopher J. Elias

**Affiliations:** 1 World HealthOrganization, Regional Office for Africa, Brazzaville, Republic of Congo; 2 World Health Organization, Geneva, Switzerland; 3 PATH, Seattle, Washington; 4 United Nations Children's Fund, New York, New York; 5 Gavi, Geneva, Switzerland; 6 Serum Institute of India Ltd, Pune; 7 Bill & Melinda Gates Foundation, Seattle, Washington

**Keywords:** group A meningococcal meningitis, vaccine, epidemic, herd protection, public/private partnership

Over the last 4 years, a remarkable public health success has unfolded in Africa. The field introduction of a new group A meningococcal conjugate vaccine, PsA-TT (MenAfriVac), has been a stunning success, with the virtual disappearance of group A meningococcal meningitis in sub-Saharan Africa. The effort began with a plea from African ministers of health to “do something” after the dreadful 1996–1997 group A meningococcal epidemic with >250 000 reported cases. The World Health Organization (WHO) convened a series of international meetings where experts recommended that a new meningococcal group A conjugate vaccine be developed for Africa.

In 2001, the Bill & Melinda Gates Foundation awarded a grant of US$70 million to create the Meningitis Vaccine Project as a partnership between PATH and WHO, with the single goal of developing, licensing, and introducing at public health scale a group A meningococcal conjugate vaccine for sub-Saharan Africa, manufactured at the Serum Institute of India, Ltd.

Using an innovative vaccine development model that paid close attention to product specifications to facilitate use of the vaccine in Africa (fewest doses required for compliance; relative stability outside cold chain) and affordability (less than US$0.50 per dose), the new vaccine, PsA-TT, was licensed by the Drug Controller General of India in December 2009 and prequalified by WHO in June 2010 [1].

The vaccine was introduced in Burkina Faso, Mali, and Niger in December 2010 and was enthusiastically accepted. By the end of that month, almost 20 million persons aged 1–29 years had been vaccinated, and the following epidemic season showed a dramatic reduction in group A meningococcal disease in all 3 countries. Vaccination campaigns have continued, and as of the end of 2014, >217 million Africans have been immunized in 15 countries. The vaccine has been shown to be safe and has generated herd protection, with control and near-elimination of group A meningococcal disease wherever it has been used [2–4].

The vaccine was further evaluated in infants and young children to prepare for its use in the Expanded Programme on Immunization (EPI), an essential step to ensure that subsequent newborn cohorts are protected and that the public health benefits continue. With study results showing safety and strong immunogenicity of a single dose at the age of 9 months and following the WHO Strategic Advisory Group of Experts on Immunization recommendations of October 2014, the vaccine is now gradually introduced as a new EPI antigen in meningitis belt countries [5].

Participants in this effort have prepared a series of manuscripts that detail the many technical, epidemiologic, and public health steps that were associated with the development, introduction, and evaluation of this vaccine. The success of the project is a testimonial to the potential of public/private partnerships to develop needed vaccines that, when introduced, can have major impact to solve important public health problems. It is our hope that the lessons learned from this endeavor will inform similar future public health initiatives.

## References

[1] LaForce FM, Okwo-Bele JM Eliminating epidemic group A meningococcal meningitis in Africa through a new vaccine. Health Aff (Millwood) 2011; 30:1049–57. 21653956 10.1377/hlthaff.2011.0328

[2] Novak RT, Kambou JL, Diomandé FV et al Serogroup A meningococcal conjugate vaccination in Burkina Faso: analysis of national surveillance data. Lancet Infect Dis 2012; 12:757–64. 22818241 10.1016/S1473-3099(12)70168-8 PMC4831863

[3] Kristiansen PA, Diomandé F, Ba AK et al Impact of the serogroup A meningococcal conjugate vaccine, MenAfriVac, on carriage and herd immunity. Clin Infect Dis 2012; 56:354–63. 23087396 10.1093/cid/cis892

[4] Daugla DM, Gami JP, Gamougam K et al Effect of a serogroup A meningococcal conjugate vaccine (PsA-TT) on serogroup A meningococcal meningitis and carriage in Chad: a community trial. Lancet 2013; 383:40–7. 24035220 10.1016/S0140-6736(13)61612-8 PMC3898950

[5] World Health Organization. Meningococcal A conjugate vaccine: updated guidance, February 2015. Wkly Epidemiol Rec 2015; 90:57–68. 25702330

